# Composites to Produce a Material with Zero Absolute Thermopower *S* = 0 or a Thermopower Switch between *S* = 0 and *S* ≠ 0

**DOI:** 10.3390/ma14195529

**Published:** 2021-09-24

**Authors:** Joachim Sonntag, Bertrand Lenoir

**Affiliations:** 1 TE Connectivity Sensors Germany GmbH, Hauert 13, D-44227 Dortmund, Germany; 2 Université de Lorraine, CNRS, IJL, F-54000 Nancy, France

**Keywords:** seebeck coefficient, seebeck coefficient standard reference, composites, Effective Medium Theory, amorphous alloys, phase separation, thermopower switch, 71.23.-k, 71.55.Jv, 72.10.Bg, 72.15.-v

## Abstract

From the theory of two-phase composites it is concluded that in the concentration dependence of the Seebeck coefficient *S* a kink can occur precisely at S=0 absolute if the two phases have different kinds of carriers, electrons and holes, and if the phase grains are spherical without preferred orientations and arranged in a symmetrical fashion. This feature, indeed found to be realized in amorphous Cr${}_{1-x}$Si${}_{x}$ thin films deposited by ion beam sputtering from Cr-Si alloy targets, can be applied to make reference standards for S=0 at room temperature and even at higher temperatures. Additionally, it may be used to design a thermopower switch between S=0 and S≠0. It is also concluded that the structure realized in any alloy during solidification does not only depend on the diffusion mobility of the atoms and on the existence of a (relative) minimum in the Gibbs’ free energy. It depends also on the fact whether this structure is compatible with the demand that (spatial) continuity of the entropy and energy flux densities and their gradients is saved during the solidification process.

## 1. Introduction

**Figure 1:**
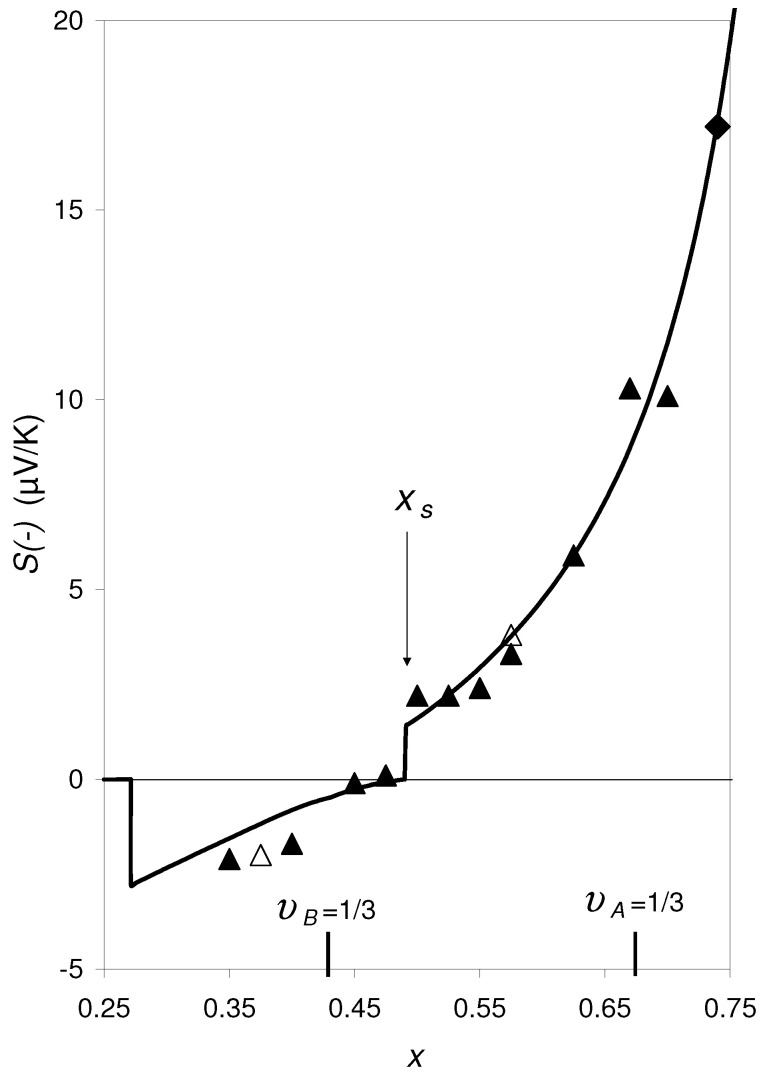
S(−) vs. *x* for *a*-Cr${}_{1-x}$Si${}_{x}$ at T=300 K calculated by Equation (1), respective Equation (9) and Equations (2)–(13) for the phases *A* (= *a*-Cr${}_{3}$Si) and *B* (= *a*-Si), where $\partial E_{C,A}/\partial T=\partial E_{V,B}/\partial T=0$ was set. This curve agrees with those of Figure A.2 in [10] for c=2.6 eV. The experimental data are taken from Gladun et al. [11] (diamonds), Weser [1] (open triangles) and Sonntag [2] (full triangles). The discontinuity in S(−) at $x=x_{s}=0.49$ corresponds to the discontinuities in Figure 2, Figure 3 and Figure 4.

Experimental data of thin *a*-Cr${}_{1-x}$Si${}_{x}$ (“*a*” in *a*-Cr${}_{1-x}$Si${}_{x}$ stands for “amorphous”) films [1,2] produced by ion beam sputtering have shown that the concentration dependence of the thermoelectric power *S* shows a discontinuity at x=0.49. Coming from x<0.49, *S* is negative and increases monotonically approaching S=0μV/K at *x* = 0.49, where *S* jumps suddenly to S=2.0μV/K (see Figure 1). With further growing *x*, *S* continues to increase monotonically. Originally, the reason for this discontinuity was not understood. Only the assumption, that *a*-Cr${}_{1-x}$Si${}_{x}$ films could be composed of two different amorphous phases (differing by short range order) gave a possible answer. Indeed, theoretical calculations based on Effective Medium Theory (EMT) have shown that these two properties, S=0μV/K and the step can be explained, if the coexistence of two different phases is assumed [3], and that such a step occurs independently of the temperature. The experimental confirmation of the presence of two amorphous phases in a series of amorphous transition-metal-metalloid alloys took place in the 1990s. Applying Raman spectroscopy, infrared absorption, extended X-ray absorption fine structure (EXAFS) and anomalous small-angle X-ray scattering (ASAXS) it has been confirmed for a number of amorphous transition metal-metalloid alloys that these indeedly consist of two different amorphous phases [4,5,6,7,8]. Regan et al. [8] found for co-sputtered *a*-W${}_{1-x}$Ge${}_{x}$, *a*-Fe${}_{1-x}$Ge${}_{x}$, *a*-Fe${}_{1-x}$Si${}_{x}$ and *a*-Mo${}_{1-x}$Ge${}_{x}$ films phase separation regions of the order of 1 nm in the growth plane and 1.5–2.0 nm in the growth direction. They could show that their measurements are in agreement with the assumption of two coexisting amorphous phases, *a*-Ge or *a*-Si, on the one side and a metallic phase with *a*-FeGe${}_{2}$, *a*-FeSi${}_{2}$, or *a*-MoGe${}_{3}$ compositions for the last three systems, respectively, on the other side. Raap et al. [7] found amorphous phases separation in co-sputtered *a*-Fe${}_{1-x}$Si${}_{x}$ films into regions of *a*-Si and an intermetallic close in composition to *a*-FeSi${}_{2}$ with ≃0.6 nm in the film plane and ≃1 nm in size in the growth direction. For the case *a*-Cr${}_{1-x}$Si${}_{x}$, the two identified amorphous phases are *a*-Cr${}_{3}$Si and *a*-Si [9]. The bonding state in these two phases differs in terms of orbital structure, sp for the metal-rich phase and $sp^{3}$ for *a*-Si.

The two properties, S=0μV/K and the discontinuity observed in *a*-Cr${}_{1-x}$Si${}_{x}$, Figure 1, deserve special interest because they can be used to produce a reference standard for the absolute thermopower (Seebeck coefficient Standard Reference material—SRM) and a thermopower switch between S=0μV/K and S≠0μV/K. SRM’s used in practice to measure the absolute thermopower of materials are generally not very accurate (see Appendix A). A decisive advantage of the proposed new SRM based on a-Cr-Si is that it can be used to set S=0μV/K very precisely.

It is important to underline that the fabrication conditions of *a*-Cr${}_{1-x}$Si${}_{x}$ films play a pivotal role on the appearence of the discontinuity in *S* at S=0μV/K. Actually, a co-sputtering approach (from a Cr target and a Si target) leads to completely different results from those found originally; both the resistivities were essentially higher and the discontinuity was not found. The difference between these two data groups was not understood. Apparently, this difference is caused by the fact that the experimental original data was produced by single deposition, i.e., for each *x* separately, whereas the experimental data produced by co-sputtering [12], i.e., during the sputtering process all the different samples with different *x* were electrically connected with each other. It is also the aim to find the reason of this difference. As it will be argued in this paper, the reason is apparently a general phenomenon, which, until now, has not yet been considered in the experimental practice, which has consequences with respect to both the dynamics of formation of the structure during production/deposition the films and, more over to the electronic transport properties as thermopower, the electrical and thermal conductivity and the Hall coefficient of the samples. It will be shown that these properties are essentially determined by the demand that (spatial) continuity of the entropy and energy flux densities and their gradients is saved during the solidification process. This has far-reaching practical consequences in the search for new materials with new properties, for which the method of “high-throughput characterization” by co-sputtering is often used today. The aim of this paper is thus twofold, first, to make known the highly exciting property of a step in *S* with S=0μV/K and its consequences (SRM and switch), and second, to recognize that co-sputtering and single-coating can give completely different results, due to the different dynamics during the layer deposition process.

As a theoretical support, the EMT formalism, which has been widely used to explain the transport properties of composites, including ceramics [13,14,15] and thermoelectrics [16,17,18], will be implemented.

The paper is structured as follows. The thermopower formula and its application are described in Section 2. Section 3 treats the effect of the carrier densities and band edges on the concentration dependence of *S*. The results of Section 2 and Section 3 and what is the cause of the difference of the two data groups described previously are discussed in Section 4 and summarized in Section 5. In the Appendix A the state of the art on SRM’s is described, and in Appendix B our EMT formula applyed in the present paper is compared with EMT formulas derived earlier by other authors.

## 2. Formulas for the Calculation of the Seebeck Coefficient

The thermopower formula applyed for the calculation of *S* vs. *x* shown in Figure 1 reads [19,20]

(1) $$\sum_{i}\upsilon_{i}\frac{\kappa_{e,i}/S_{i}-\kappa_{e}/S}{\kappa_{e,i}/S_{i}+2\kappa_{e}/S}=0,$$

where *S* and $\kappa_{e}$ are the Seebeck coefficient and the electronic contribution to the thermal conductivity of the composite, κ. $S_{i}$, $\kappa_{e,i}$ and $\upsilon_{i}$ are the corresponding parameters and the volume fraction of the phase *i* (A,B,…). The thermopower formula Equation (1) is derived on basis of the Effective Medium Theory (EMT).

For the derivation of Equation (1) the following assumptions were made: The alloy is a composite consisting of two different phases which form spherical phase grains, randomly arranged and in a symmetrical fashion. This assumption is only an approximation, but we believe that this is a good description of the principle behavior of the current flow densities through the composite consisting only of amorphous phase grains. There are also attempts to consider deviations from a spherical shape in the formulas, for instance the Generalized Effective Medium Theory (GEMT), based on a phenomenological model with the addition of elements of the percolation theory to the EMT. This idea was applied by Vaney et al. [18] to *S* for crystalline composites.

Each phase is characterized by its own transport coefficients. At the boundary face between a single phase grain and the surrounding ‘effective medium’ continuity of the current densities and potentials and their gradients are saved, and the additional condition $J=J_{i}=0$ is to be fulfilled. $J_{i}$ and *J* are the electrical current density in a single phase grain and the surrounding ‘effective medium’, respectively.

*S* vs. $\upsilon_{i}$ can be calculated by Equation (1) if $S_{i}$, $\kappa_{e,i}$ and $\kappa_{e}$ are known. $S_{i}$ can be calculated by [10]

(2) $$S_{i}=S_{i}^{0}+\frac{1}{\left|e\right|}\frac{d\mu }{dT},$$

where μ is the electrochemical potential, and *T* the absolute temperature. $S_{i}^{0}$ and $\frac{1}{\left|e\right|}\frac{d\mu }{dT}$ are the “scattering term” in the phase *i* and the “thermodynamic term”. The “thermodynamic term” is identical for all the phases in the composite.

For metallic phases, $S_{i}^{0}$ and $\kappa_{e,i}$ are given by

(3) $$S_{i}^{0}=\frac{\pi^{2}k_{B}^{2}T\left(1+r_{i}\right)}{3e_{i}E_{F,i}},$$

(4) $$\kappa_{e,i}=\frac{16\pi^{3}}{9}\frac{m_{i}L_{i}E_{F,i}}{h^{3}}k_{B}^{2}T,$$

following from the Boltzmann transport equation (BTE) in the approximation of nearly free electrons (NFE). |e| is the elementary charge; $e_{i}=-\left|e\right|$ and +|e| for electrons and holes, respectively. $m_{i}$ and $L_{i}$ are the effective mass and the mean free path, respectively, in the phase *i*. $r_{i}$ characterizes the energy dependence of $L_{i}$ according to $L_{i}\propto E^{r_{i}}$. *h* is the Planck’s constant, and $k_{B}$ the Boltzmann constant. $E_{F,i}$ is the Fermi energy given by

(5) $$E_{F,i}=\frac{h^{2}}{8m_{i}}(\frac{3}{\pi }{)}^{2/3}n_{i}^{2/3}$$

for a phase *i* with electron conductivity having the electron density $n_{i}$. The approximation in Equation (4) is surely a good one for metallic phases, if the phases form macroscopic clusters. The formula for κ (total thermal conductivity) derived by Odelevskii [21] reads

(6) $$\sum_{i}\upsilon_{i}\frac{\kappa_{i}-\kappa }{\kappa_{i}+2\kappa }=0.$$

Therefore we assume that

(7) $$\sum_{i}\upsilon_{i}\frac{\kappa_{e,i}-\kappa_{e}}{\kappa_{e,i}+2\kappa_{e}}=0$$

is valid as well.

## 3. The Effect of the Carrier Densities and Band Edges on S vs. $\upsilon_{B}$

Let us consider a two-phase composite where the phase *A* has electron conductivity with $n\equiv n_{A}$, whereas the phase *B* has hole conductivity with the hole density *p*, for which the Fermi energy reads

(8) $$E_{F,B}=\frac{h^{2}}{8m_{B}}(\frac{3}{\pi }{)}^{2/3}p^{2/3}$$

(characterized by Figure 1c in [3]).

**Figure 2:**
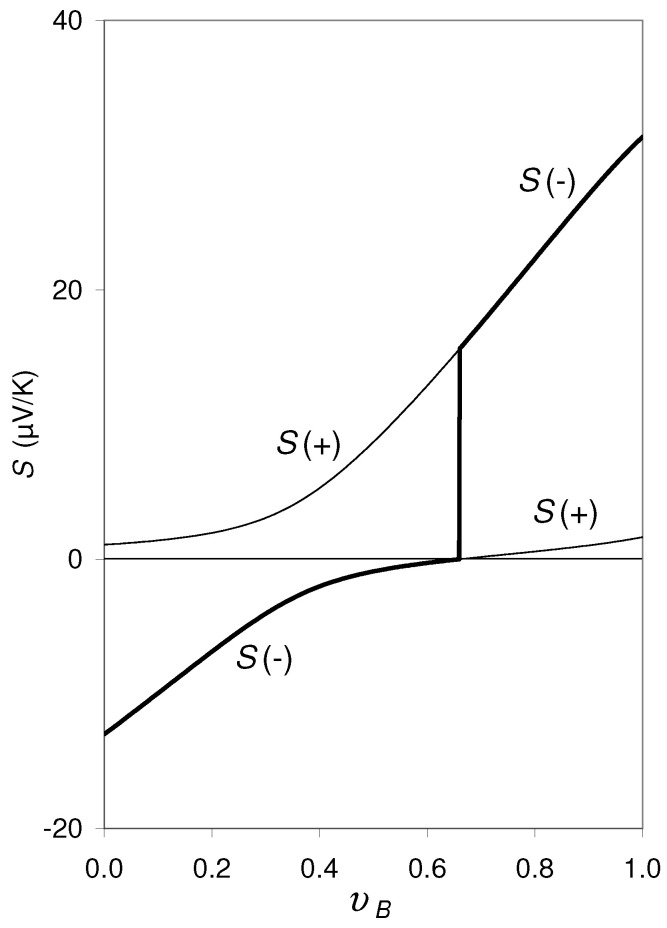
S(−) (bold line) and S(+) (thin line) vs. $\upsilon_{B}$ calculated by Equations (2)–(13) for a hypothetical composite with $S_{A}^{0}=-13.0\;\mu$V/K, $S_{B}^{0}=+1.5\;\mu$V/K, $\kappa_{e,A}=8.5$ mW/cmK, and $\kappa_{e,B}=12.7$ mW/cmK (corresponding to $n=10^{22}$ cm${}^{-3}$ and $p=2\times 10^{22}$ cm${}^{-3}$ and T=300 K, where dμ/dT, Equation (10), is calculated for $\partial E_{C,A}/\partial T=\partial E_{V,B}/\partial T=\partial \varphi_{i}/\partial n_{i}=0$).

Equation (1) has two solutions, S(+) and S(−),

(9) $$S=S\left(\pm \right)=\frac{4\kappa_{e}}{ℜ\pm \sqrt{{ℜ}^{2}+8\left(\kappa_{e,A}/S_{A}\right)\left(\kappa_{e,B}/S_{B}\right)}}$$

with $ℜ=\left(3\upsilon_{A}-1\right)\kappa_{e,A}/S_{A}+\left(3\upsilon_{B}-1\right)\kappa_{e,B}/S_{B}$ and $\upsilon_{A}+\upsilon_{B}=1$. In Figure 2 an example of calculation is shown for a hypothetical composite with $S_{A}^{0}=-13.0\;\mu$V/K, $S_{B}^{0}=+1.7\;\mu$V/K, $\kappa_{e,A}=8.5$ mW/cmK, and $\kappa_{e,B}=12.7$ mW/cmK. These transport parameters correspond to $n=10^{22}$ cm${}^{-3}$ and $p=2\times 10^{22}$ cm${}^{-3}$ if calculated by Equations (3)–(5) and Equation (8) with $m_{A}=m_{0}$, $m_{B}=0.2\times m_{0}$, $r_{i}=2$ and $L_{i}=4/\pi \times d_{i}$ ([22], p. 348) at T=300 K. $m_{0}$ is the bare electron mass. $d_{i}$ is the interatomic distance in the phase *i* ($d_{A}=0.25$ nm and $d_{B}=0.234$ nm). For calculating dμ/dT in Equation (2),

(10) $$\frac{d\mu }{dT}=\frac{\partial E_{C,A}}{\partial T}+\frac{\partial \mu_{A}^{0}}{\partial T}-\frac{\frac{\partial \mu_{A}^{0}}{\partial T}+\frac{\partial \mu_{B}^{0}}{\partial T}+\frac{\partial E_{C,A}}{\partial T}-\frac{\partial E_{V,B}}{\partial T}}{1+\frac{\upsilon_{A}\left(\frac{\partial \mu_{B}^{0}}{\partial p}-\left|e\right|\frac{\partial \varphi_{B}}{\partial n_{B}}\right)}{\upsilon_{B}\left(\frac{\partial \mu_{A}^{0}}{\partial n}-\left|e\right|\frac{\partial \varphi_{A}}{\partial n}\right)}}$$

has been applied with

(11) $$\frac{\partial \mu_{i}^{0}}{\partial T}=-\frac{\pi^{2}k_{B}^{2}T}{6E_{F,i}},$$

(12) $$\frac{\partial \mu_{A}^{0}}{\partial n}=\frac{2E_{F,A}}{3n},$$

(13) $$\frac{\partial \mu_{B}^{0}}{\partial p}=\frac{2E_{F,B}}{3p}$$

(following from the Fermi–Dirac-statistics), where the contributions by the band edges ($E_{C,A},E_{V,B}$) and those of the electrostatic potential ($\varphi_{i}$) to dμ/dT were still neglected, i.e., $\partial E_{C,A}/\partial T=\partial E_{V,B}/\partial T=\partial \varphi_{i}/\partial n_{i}=0$ was set. $\varphi_{i}$ and $\mu_{i}^{0}$ are the electrostatic potential and the chemical potential, respectively, in the phase *i*. $E_{C,A}$ and $E_{V,B}$ are the band edges of the conduction band (CB) in the phase *A* and the valence band (VB) in the phase *B*, respectively. Note that $dp=-dn_{B}$. With this dμ/dT, $S_{i}$ and S(±) are calculated by Equation (2) and Equation (9), respectively. The result is shown in Figure 2: At $\upsilon_{B}=0.63$ there is a discontinuity in the S(−) and S(+) curves, where the lower kink occurs exactly at S(−)=S(+)=0.

**Figure 3:**
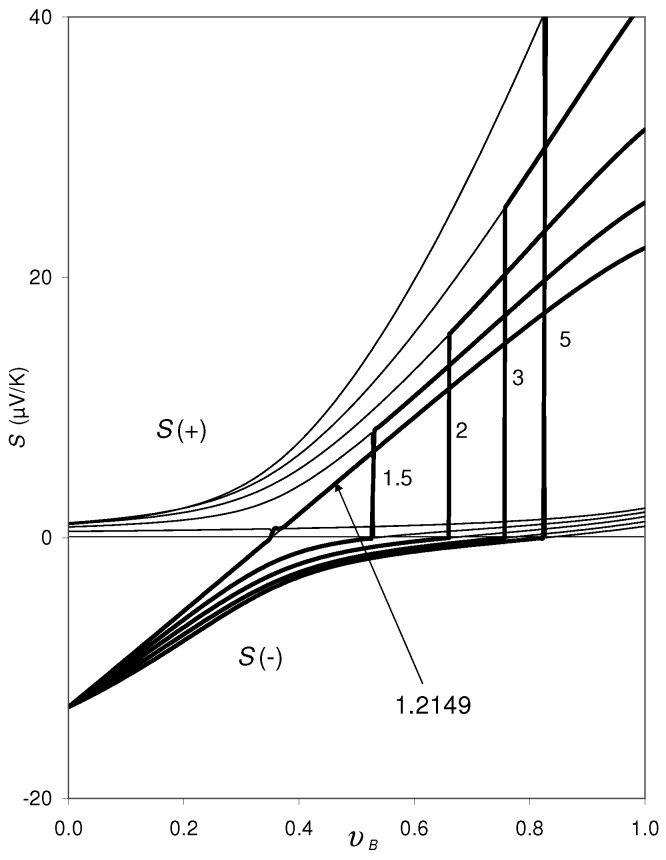
Same as Figure 2, for varying values of *p*: p=1.5,2,3, and 5 (in units of $10^{22}$ cm${}^{-3}$), while $\partial E_{C,A}/\partial T=\partial E_{V,B}/\partial T=\partial \varphi_{i}/\partial n_{i}$ = 0 is set. The discontinuity in the curves shifts to lower $\upsilon_{B}$ as *p* decreases. For $p<1.2149\times 10^{22}$ cm${}^{-3}$ (and $n=10^{22}$ cm${}^{-3}$), Equation (9) does not have real solutions for the entire concentration range. For $p>1.2149\times 10^{22}$ cm${}^{-3}$ the lower kink of S(±) at the discontinuity occurs always at S(−)=S(+)=0.

An analogous result is also obtained for other values of *n* and *p*. Figure 3 shows S(±) vs. $\upsilon_{B}$ for different values of *p*, while *n* is hold constant, $n=10^{22}$ cm${}^{-3}$, and the other parameters $m_{i}$, $r_{i}$, $L_{i}$ are identical to those applied in Figure 2. With decreasing the value of *p*, the size of the discontinuity (the distance between the upper and lower kinks) decreases continuously, until it disappears completely at a critical value of *p* and a gap opens in the concentration range where there are no real solutions S(±), because the square root in Equation (9) becomes imaginary. For the example composite shown in Figure 3, this critical value is $p_{crit}=1.2149\times 10^{22}$ cm${}^{-3}$: For $p\leq p_{crit}$, Equation (9) has no longer real solutions for the entire concentration range.

If *n* is varied, while *p*, $m_{i}$, $r_{i}$ and $L_{i}$, are identical to those applied in Figure 2, we get a similar result: Here the critical value is $n_{crit}=1.24\times 10^{22}$ cm${}^{-3}$. For $n\geq n_{crit}$ (and $p=2\times 10^{22}$ cm${}^{-3}$), Equation (9) has no longer real solutions for the entire concentration range.

**Figure 4:**
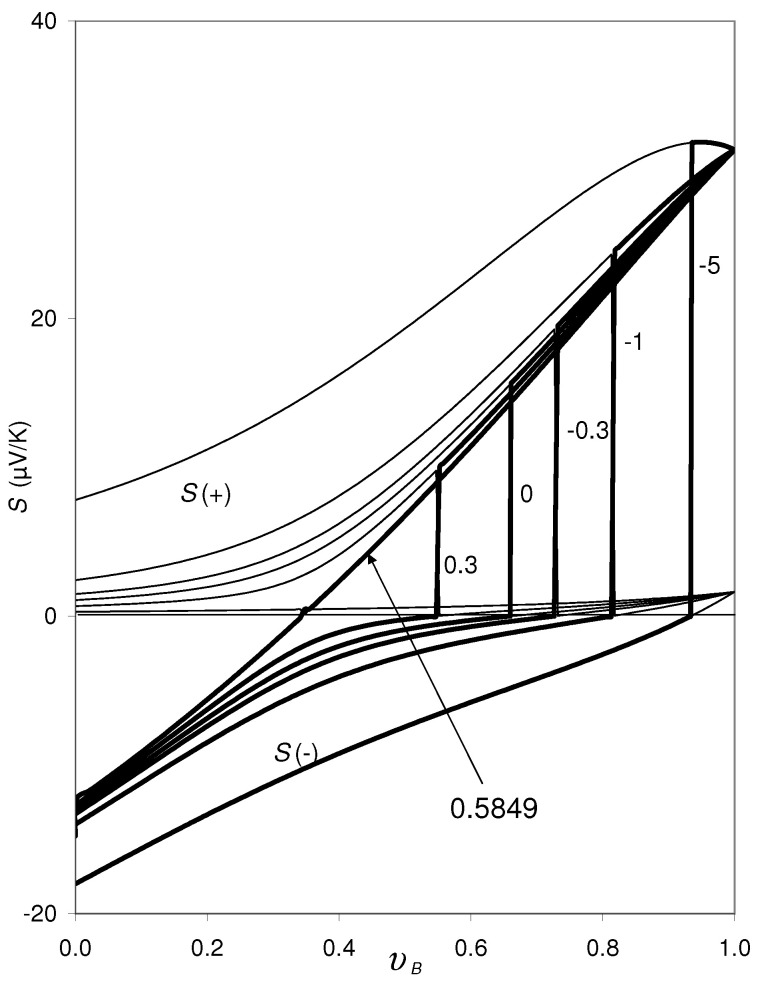
Same as Figure 2, for varying values of $\partial E_{C,A}/\partial T$: $\partial E_{C,A}/\partial T=-5\;,\;-1,\;-0.3,\;0,\;0.3$, and 0.5849 (in units of $10^{-6}$ eV/K), while $\partial E_{V,B}/\partial T$ = 0 and $\partial \varphi_{i}/\partial n_{i}$ = 0 is set. The discontinuity in the curves shifts to lower $\upsilon_{B}$ as $\partial E_{C,A}/\partial T$ increases. For $\partial E_{C,A}/\partial T>0.5849$, Equation (9) does not have real solutions for the entire concentration range. For $\partial E_{C,A}/\partial T<0.5849$, the lower kink of S(±) at the discontinuity occurs at S(−)=S(+)=0, always.

Until now fixed band edges have been assumed. However, the thermopower of the phases $S_{i}$, Equation (2), depend on dμ/dT, and dμ/dT depends on $\partial E_{C,A}/\partial T$ and $\partial E_{V,B}/\partial T$, according to Equation (10). In Figure 4 the effect of $\partial E_{C,A}/\partial T$ on the solutions of Equation (9) is shown. For this variation, in Equation (2) $\partial E_{V,B}/\partial T=\partial \varphi_{i}/\partial n_{i}$ = 0 is set, and the other parameters are identical to those applied in Figure 2. The discontinuities in the S(−) curve (bold line) and the S(+) curve (thin line) are shifted to lower $\upsilon_{B}$ as $\partial E_{C,A}/\partial T$ increases. Here the critical value is ${\left(\partial E_{C,A}/\partial T\right)}_{crit}=0.5849\times 10^{-6}$ eV/K. For $\partial E_{C,A}/\partial T>{\left(\partial E_{C,A}/\partial T\right)}_{crit}$ there are no real solutions for the entire concentration range $0<\upsilon_{B}<1$.

A variation of $\partial E_{V,B}/\partial T$ has a similar effect as a variation of $\partial E_{C,A}/\partial T$. There the critical value is ${\left(\partial E_{V,B}/\partial T\right)}_{crit}=1.8967\times 10^{-6}$ eV/K. For $\partial E_{V,B}/\partial T>{\left(\partial E_{V,B}/\partial T\right)}_{crit}$ there are no real solutions for the entire concentration range $0<\upsilon_{B}<1$.

When we vary the parameters *n*, *p*, $\partial E_{C,A}/\partial T$ and $\partial E_{V,B}/\partial T$, the lower kink of S(±) at the discontinuity occurs at S(−)=S(+)=0, as long as we are not beyond the critical values mentioned.

The two S(−) curves in Figure 4 for ${\left(\partial E_{C,A}/\partial T\right)}_{crit}=0.5849\times 10^{-6}$ eV/K and $\left(\partial E_{C,A}/\partial T\right)=+0.3\times 10^{-6}$ eV/K are very similar to the experimental data for amorphous *a*-Cr${}_{1-x}$Si${}_{x}$ thin films, deposited under different deposition conditions: For the *a*-Cr${}_{1-x}$Si${}_{x}$ films sputtered from different Cr${}_{1-x}$Si${}_{x}$ targets in *single* manufacturing processes, there is a significant step in the *S* versus concentration dependence as shown in Figure 1, whereas *a*-Cr${}_{1-x}$Si${}_{x}$ films sputtered from a Cr target and a Si target arranged separately from each other (co-sputtering) does not show such a step [12]. The essential difference between these two methods of deposition is the fact, that in the co-sputtered films there is a common dμ/dT for all the *x* realized during the deposition run, whereas for the films sputtered from different Cr${}_{1-x}$Si${}_{x}$ targets, dμ/dT is different for the different *x*. Between these two series of *a*-Cr${}_{1-x}$Si${}_{x}$ films there are also considerable differences regarding their specific resistivity ρ versus *x* dependences ([23], Figure 7 therein).

## 4. Discussion

As shown in Section 3 for the hypothetical composite, S(−) and S(+) approach always the value “0” at the lower kink of the discontinuity provided that the carriers in the two phases have different signs, electrons and holes (the concentration where this kink occurs will be noted $\upsilon_{B}=\upsilon_{B,s}$ and $x=x_{s}$ for the corresponding atomic concentration). This property can be used to construct “Seebeck coefficient Standard Reference with 0 μV/K absolute”. In order to use this property, the following questions are still to be answered:

(1) Does the experimental Seebeck curve $S_{exp}$ vs. *x* (respectively $S_{exp}$ vs. $\upsilon_{B}$) of a real two-phase alloy follow one of the solutions, S(−) or S(+), or does it follow a smooth curve changing between S(−) to S(+) at the discontinuity? (see Figure 2)
(2) If $S_{exp}$ vs. *x* follows one of the solutions, S(−) or S(+), does then the lower kink of the discontinuity in the $S_{exp}$ vs. *x* curve occur at $S_{exp}=0$?
(3) Which effect do the electrostatic potentials have on the discontinuity in S(±)?
(4) Which meaning or consequence has the fact that Equation (1) (respectively Equation (9)) does not have real solutions for the entire concentration range if the carrier densities or the band edge shifts are beyond the critical values specified earlier?

Let us start with the point (1). If the thermopower changed between S(−) and S(+) at the discontinuity, no discontinuity in $S_{exp}$ vs. *x* is expected experimentally. However, the experimental data in Figure 1 apparently follow the calculated S(−) curve on both sides of the discontinuity at $x_{s}=0.49$, i.e., they does not cross over from S(−) to S(+) or vice versa. Contrarily to that, for the two limiting cases “$\upsilon_{B}=0$” and “$\upsilon_{B}=1$” it follows from Equation (9) with Equation (7) that S(+) is the physical solution.

Let us move now to the second question, (2): As can be seen in Figure 1, at the lower kink of the S(−) curve at $x_{s}$, S(−)=0. Actually, coming from the left-hand side of this discontinuity, the experimental thermopower data approach zero precisely at the lower kink at $x_{s}$. This experimental finding corresponds to the calculated S(−) curves for the hypothetical composite specified in Section 2 drawn in Figure 2 and Figure 3. (Note that $\upsilon_{B}$ increases monotonically with *x* provided that the phase compositions, $x_{A}$ and $x_{B}$, are constant.)

We emphasize that the jump in the thermopower at S=0 is not the result of approximations. It also does not depend on the choice of the model applied for calculation of the transport coefficients of the phases *A* and *B* (“parabolic band model with an effective mass and power law scattering rates”). The jump at S=0 always follows purely mathematically from Equation (1), independent of the chosen numbers for $S_{i}$ and $\kappa_{e,i}$, if Equation (1) has a mathematical solution at all for S(−) and S(+).

It is clear that if one of the two phases is present as separate islands, the band model can no longer be applied to this phase. This is true for $\upsilon_{B}<1/3$ for phase *B* and, on the other hand, also for $\upsilon_{A}<1/3$ for phase *A*. In amorphous composites this limit, which separates “island structure” and “continuous structure” from each other, is very precisely at $\upsilon_{B}=1/3$ with respect to phase *B*. The same is true for phase *A*. Details to this problem are discussed in detail in [19].

**Figure 5:**
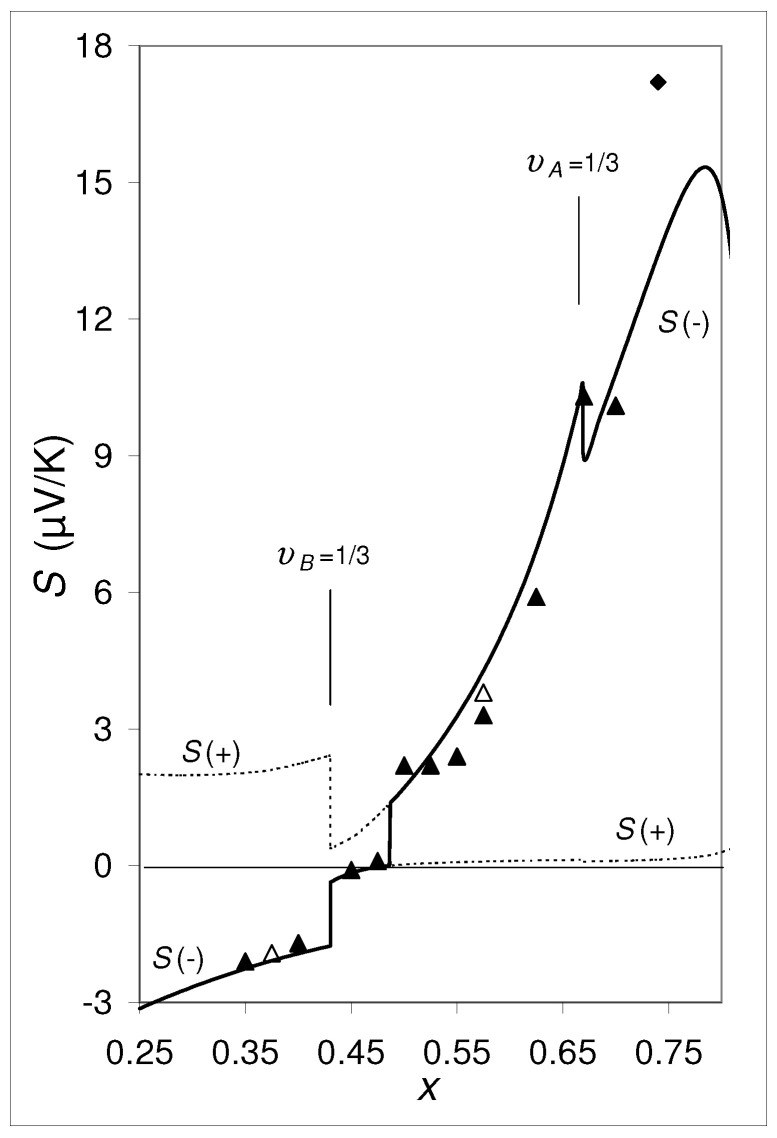
Same as Figure 1, where however $S_{B}=0$ and $\kappa_{e,B}=0$ are set for $\upsilon_{B}<1/3$ and $S_{A}=0$ and $\kappa_{e,A}=0$ are set for $\upsilon_{A}<1/3$. For $\upsilon_{i}<1/3$ the phase *i* exists as islands separated from each other. (The curve for S(−) agrees with that obtained by Sonntag [19]).

In Figure 5, the same curve S(−) of Figure 1 is shown once more, however with the difference that $S_{B}=0$ and $\kappa_{e,B}=0$ are set for $\upsilon_{B}<1/3$, and $S_{A}=0$ and $\kappa_{e,A}=0$ are set for $\upsilon_{A}<1/3$. For $\upsilon_{i}<1/3$ the phase *i* exists as islands separated from each other, where the electronic bands in the phase *i* are not formed. The curve S(−) in Figure 5 agrees relatively well with the experimental data. It is noteworthy that at $\upsilon_{A}=1/3$ and at $\upsilon_{B}=1/3$ two more steps appear, which are also reflected in the experimental data.

If Equation (1) does not provide a mathematical solution, we conclude that the underlying physical model (the “EMT”-assumption that “the phase grains are spherical with no preferred orientations and arranged in a symmetrical manner”) is not a good approximation to describe the structure of the alloy. More on this in the next points, (3) and (4) that are treated in the following paragraphs.

The electrostatic potential affects the thermopower of a composite, but does not affect it in a homogeneous material [10]. For the example alloy *a*-Cr${}_{1-x}$Si${}_{x}$ it is shown that the discontinuity in S(−) shifts to larger $\upsilon_{B}$ as the electrostatic parameter *c* increases and one may conjecture that such a situation where Equation (1) does not have solutions for the entire concentration range is not realized in the nature, because the electrostatic potentials act contrarily to the effect of $\partial E_{C,A}/\partial T$ (respectively $\partial E_{V,B}/\partial T$) [10]. For instance, assuming that $\partial E_{C,A}/\partial T>0$, then a temperature depending electron transfer occurs from the phase *A* to the phase *B* leading to an increase of the electrostatic potential difference between the phases counterbalancing the effect of $\partial E_{C,A}/\partial T$ on the solutions of Equation (1).

However, such a counterbalancing is surely incompletely, and one can conjecture that situations are possible in nature (i.e., experimental conditions) where Equation (1) does not have solutions for the entire concentration range. For concentration ranges, where Equation (1) does not have a mathematic solution, we speculate that the structure and arrangement of the phase grains realized is different from that assumed for the derivation for Equation (1), for instance that the phase grains are not spheric or/and arranged in an asymmetrical fashion or/and there are preferred orientations of them. The reason is the following: Equation (1) has been derived under the condition that at the boundary face between a single spherical phase grain and its surroundings, (spatial) continuity of the entropy-flux density, $J_{S}$, and its gradient is fulfilled [19]. This condition must also be fulfilled during the solidification process of the alloy. If Equation (1) does not have a solution (for a certain *x*), then the condition of continuity of $J_{S}$ at the boundary faces between the different phases cannot be fulfilled leading to the fact that such a structure as specified cannot be realized. Instead, another atomic structure is favored, where the continuity of $J_{S}$ and its gradient can be fulfilled. This other atomic structure can be, for instance, characterized by non-spheric phase grains or other values for $x_{A}$ (and $x_{B}$) corresponding to a situation where the phase compositions become a function of *x*. Saving continuity of $J_{S}$ and its gradient, the structure of the alloy is matched at the especial conditions prevailing during the solidification of the alloy. This discussion can also be executed considering the energy flux density $J_{E}$ and its gradient.

In other words, the structure realized in any alloy does not only depend on the diffusion mobility of the atoms and whether there exists a (relative) minimum in the Gibbs’ free energy. The structure realized depends also on the fact whether it is compatible with the demand that continuity of the entropy and energy flux densities and their gradients is saved during the solidification process.

This conclusion is a consequence of the Gibbs equation and its conditions of validity. Gibbs equation reads

(14) $$dU=TdS+\sum_{i}\mu_{i}^{0}dn_{i},$$

where $n_{i}$ is the particle density of species *i*. *U* is the internal energy density, S is the entropy density, and $\mu_{i}^{0}$ is the chemical potential of the *i*th species present in the system. Equation (14) holds both for electronic carriers and atoms and ions [24,25]. Introducing

(15) $$E=U+\varphi_{s}q$$

and considering the Gibbs–Duhem relation, which is applicable to the function *U*, it follows

(16) $$S\frac{\partial T}{\partial t}+\sum_{i}n_{i}\frac{\partial \mu_{i}^{0}}{\partial t}=0,$$

where *t* is the time. With the electrochemical potential $\mu_{i}$ we get

(17) $$\frac{\partial E}{\partial t}=T\frac{\partial S}{\partial t}+\sum_{i}\mu_{i}\frac{\partial n_{i}}{\partial t}+q\frac{\partial \varphi_{s}}{\partial t},$$

where $\varphi_{s}$ is the electrostatic potential. *q* is the net charge density with $q=\sum_{i}q_{i}$. $q_{i}$ is the net charge density of the species *i*. Equation (17) is the basic formula for deriving the transport equations to describe the electronic transport processes, but also for diffusion processes in solids, expressed by the transport coefficients, to bring the system into equilibrium. Equation (17) is based on the continuity of the energy and entropy flow densities and their derivations according to place and time so that they do not break off. For our above conclusion it is now important under which conditions Equation (17) holds. “From an empirical viewpoint, use of the Gibbs equation is justified on the basis that, except for turbulence and shock-wave phenomena, it leads to excellent agreement with experiment. Therefore, we take the view that Equation (17) remains applicable, as long as local deviations from equilibrium are sufficiently small.” (quoted from Harman and Honig [24], p. 10 therein). One can now object that this condition, that the local deviations from the equilibrium state during the layer deposition, are not small, because the process of layer growth is certainly far away from an equilibrium state. However, this means nothing other than that the resulting phase distribution of the growing layer on the substrate can more or less deviate from an uniform distribution and that also the shape of the phase grains can more or less deviate from a spherical shape. This means that the assumption for the derivation of the EMT approximation made for Equation (1), “the phase grains are spherical with no preferred orientations and arranged in a symmetrical fashion” is not or only partially fulfilled. This is all the more serious if there are not only microscopic concentration gradients on the substrate (single target deposition), but a macroscopic, pronounced concentration gradient over the entire substrate in the horizontal direction (co-sputter deposition). Because Equation (17) is also the basis for the derivation of Equation (1), it cannot apply if the deposition conditions deviate too much from the equilibrium.

A situation, where Equation (1) has no solution, is equivalent to a situation, where the demand of this continuity of the entropy and energy flux densities and their gradients cannot be fulfilled by such a structure as assumed leading to a modified grain structure and structural arrangement.

Such a modified structure could be for example such that the phase grains are not spherical or such that there are additional holes or spaces at the phase boundaries. In this last case, the resistivity of the alloy is expected to be higher than without these defects.

For the assumption of holes or spaces speeks the fact, that the resistivities of the films produced by co-sputtering are essentially higher than the films produced by single sputtering. The question, which structure is actually realized, can be answered by specific structural investigations.

**Figure 6:**
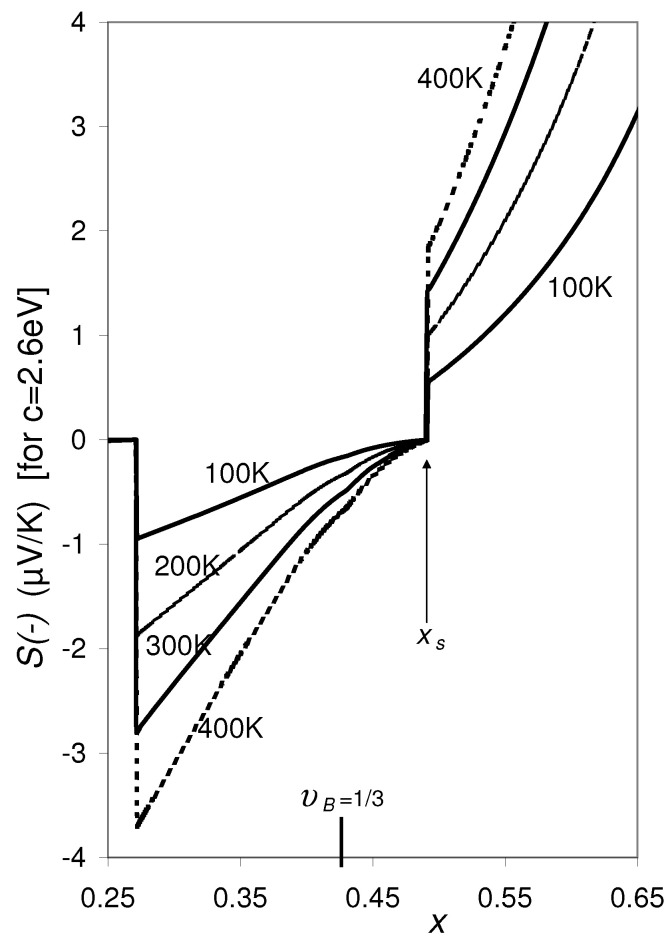
Same as Figure 1, however S(−) vs. *x* calculated for different temperatures: *T* = 100, 200, 300, and 400 K. The discontinuity at x=0.27 disappears if $S_{B}^{0}=0$ and $\kappa_{e,B}=0$ is set for $\upsilon_{B}<1/3$, which is a better approximation than given by Equation (3) and Equation (4), because the phase B does no longer form a macroscopic cluster through the alloy; at $\upsilon_{B}=1/3$ the energy spectrum changes from a quasi-continuous spectrum ($\upsilon_{B}>1/3$) to a discrete energy spectrum ($\upsilon_{B}<1/3$) typical for separate phase grains [19].

Independently of this open question, we state that for all the cases considered in Figure 2, Figure 3, Figure 4, Figure 5 and Figure 6, the lower kink at the discontinuity in the calculated S(−) curves is exactly at S(−)=0, provided that the critical values for *n*, *p*, $\partial E_{C,A}/\partial T$, $\partial E_{V,B}/\partial T$ are not yet exceeded.

### Thermopower Switch

**Figure 7:**
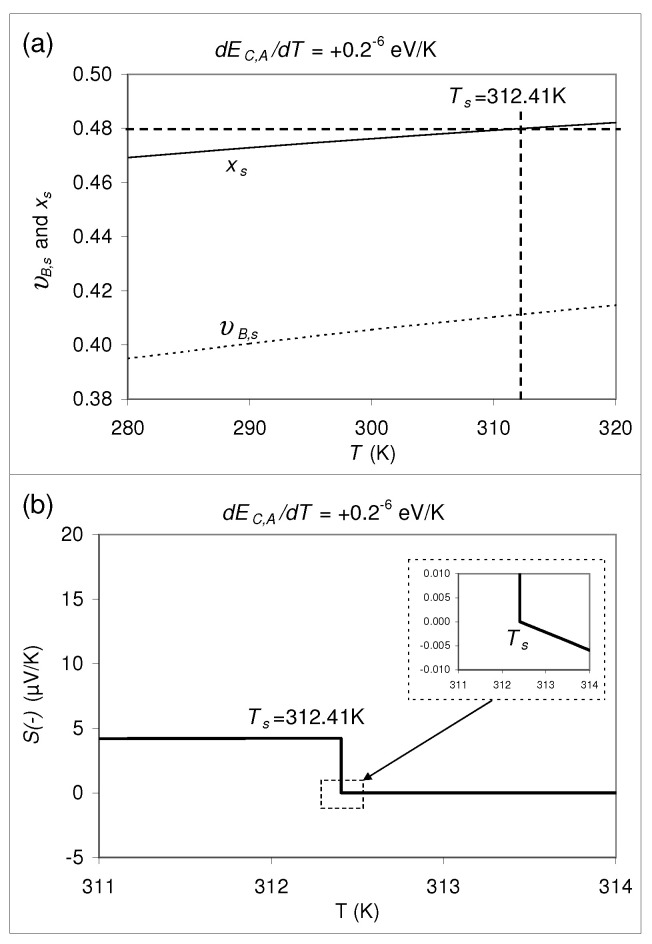
(**a**) $x_{s}$ and $\upsilon_{B,s}$ vs. *T* calculated for the example of Figure 4 with $\partial E_{C,A}/\partial T=+0.2\times 10^{-6}\;$ eV/K. For x=0.48, $T_{s}=312.41K$. (**b**) S(−) vs. *T* for x=0.48. The inset shows the same data with a better resolution. At the lower kink of the discontinuity, S(−)=0 precisely; S(−) decreases with increasing *T* very slowly at a rate of −0.0037 (μV/K) per 1 K.

**Figure 8:**
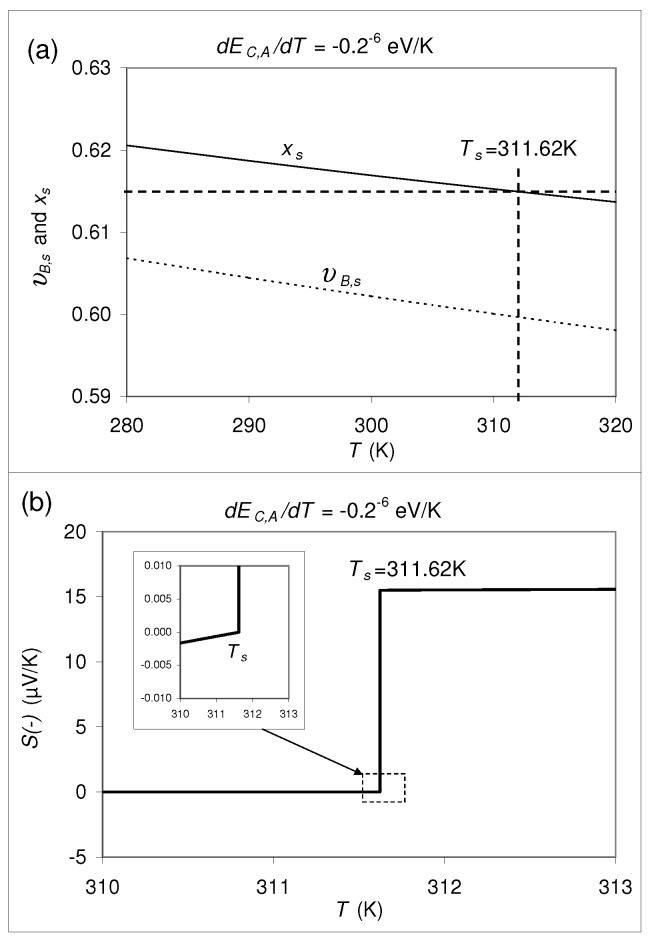
(**a**) Same as Figure 7, but with $\partial E_{C,A}/\partial T=-0.2\times 10^{-6}\;$ eV/K. For x=0.615, $T_{s}=311.62\;K$. (**b**) S(−) vs. *T* for x=0.615. The inset shows the same data with a better resolution. At the lower kink of the discontinuity, S(−)=0 precisely; S(−) increases with increasing *T* very slowly at a rate of 0.001 (μV/K) per 1 K.

As shown in Figure 1, coming from x<0.49 and increasing *x*, at room temperature the thermopower jumps at $x=x_{s}=0.49$ from S=0μV/Kto *S* = 2.0 μV/K. Now let us ask whether or not the value of $x_{s}$ is temperature dependent? By applying Equation (1) one can show, that the calculated value of $x_{s}$ is actually a function of *T*. This is shown for two choosen examples in Figure 7 and Figure 8, where several values are assumed for the physical parameters which are contained in the formulas summarized in Section 2 and Section 3. Indeedly, for both cases there is a jump in *S* vs *T*. That is why, we assume that such a temperature dependence can also be expected for real composites or nanocomposites.

By choosing of a suitable composition *x* very close to $x_{s}$, we have the possibility to produce a reference standard for the Seebeck coefficient *S*, where S=0μV/K absolute. Until now, such (theoretically established) reference standards for S=0μV/K were restricted to superconductors below the critical temperature.

For *a*-Cr${}_{1-x}$Si${}_{x}$, S(−)=0 at $x_{s}$, independently of the temperature, as shown in Figure 6. This finding is especially noteworthy, because (contrarily to that) the thermopower in a homogeneous metal depends on *T*. For the case of temperature independent band egde, in a homogeneous “NFE”-metal, *S* depends even linearly on *T* (in correspondence to [10], Equations (38) and (39) therein).

$x_{s}$ itself (characterized by S(−)=0), depends, however, on temperature if $\partial E_{C,A}/\partial T\neq 0$ or $\partial E_{V,B}/\partial T\neq 0$, i.e., for two different temperatures the values for $x_{s}$ are different. Only for the hypothetical special case that $\partial E_{C,A}/\partial T=\partial E_{V,B}/\partial T=0$, $x_{s}$ turns out to be independent of *T*. This fact seems to be a disadvantage for a realization of a highly precise reference standard with S=0. On the other hand, it can also be an advantage: By choice of an appropriate composition *x* close to $x_{s}$ one could produce a “thermopower switch”, which swiches between S=0μV/K and a finite S≠0 at a certain temperature, the switching temperature $T_{s}$. If this “switching property” is actually realized for a given *x* close to $x_{s}$, the value S=0μV/K could be adjusted arbitrarily precisely by variation of *T* approaching $T_{s}$, so that this material could serve, after all, as a very precise reference standard for S=0μV/K absolutely. While this formal discussion is, to a certain extent, speculative, let us calculate such a “thermopower switch” for the hypothetical composite specified in Section 2. In Figure 7 and Figure 8 such a “swiching” property is shown for $\partial E_{C,A}/\partial T=+0.2\times 10^{-6}\;$ eV/K and $-0.2\times 10^{-6}\;$ eV/K, respectively, where simultaneously $\partial E_{V,B}/\partial T=\partial \varphi_{i}/\partial n_{i}=0$ was set. In the Figure 7a and Figure 8a, the $\upsilon_{B,s}$ and $x_{s}$, related by

(18) $$x_{s}=\frac{x_{A}N_{A}\left(1-\upsilon_{B,s}\right)+x_{B}N_{B}\upsilon_{B,s}}{N_{A}\left(1-\upsilon_{B,s}\right)+N_{B}\upsilon_{B,s}},$$

are drawn vs. *T*. $N_{i}$ and $x_{i}$ are the atomic density and the atomic concentration in percent in the phase *i*, respectively, which were set to be $N_{A}$ =7.9 ×$10^{22}$ cm${}^{-3}$, $N_{B}$ = 5.0 × 10${}^{22}$ cm${}^{-3}$, $x_{A}=0.25$, and $x_{B}=1.00$.

According to Figure 7a, a composite with $\partial E_{C,A}/\partial T=+0.2\times 10^{-6}$ eV/K and x=0.48 would show a switching temperature of $T_{s}$ = 312.41 K with a switching property as indicated in Figure 7b. According to Figure 8a, a composite with $\partial E_{C,A}/\partial T=-0.2\times 10^{-6}$ eV/K and x=0.615 would show a switching temperature of $T_{s}$ = 311.62 K with a switching property as indicated in Figure 8b. In both Figure 7b and Figure 8b, at the lower kink of the discontinuity, S(−)=0 precisely. As can be seen in the insets of Figure 7b and Figure 8b, on the side where S(−) is close to 0, S(−) changes very slowly with *T* with rates of −0.0037 (μV/K) per 1 K and 0.001 (μV/K) per 1 K, respectively.

However, we emphasize that such a “switching property” of *S* is a fictitious result following from an application of the thermopower formula Equation (1) to the two-phase composite specified in Section 2 with the additional property that $E_{C,A}$ depends on *T*. Note that for the calculations shown in Figure 7 and Figure 8 the effect of the electrostatic potentials has been neglected. Taking into account these contributions, the “switching properties” are expected to be modified, and there remains the open question whether such a hypothetical discontinuity occurs really in the *S* vs. *T* dependence or whether the effect of the electrostatic potential counterbalances this effect of band edge shift. Therefore, it is not at all clear whether or not there are real composites with such a “switching property”.

Suitability of *a*-Cr${}_{1-x}$Si${}_{x}$ films as reference standard: Amorphous alloys are generally in a relatively unstable state. In contrast to this general experience, for not too small *x*, *a*-Cr${}_{1-x}$Si${}_{x}$ thin films, adequately annealed below the crystallization temperature $T_{k}$, can be very stable as long as the temperature of application is essentially lower than $T_{k}$. This high stability is assumed to be caused especially by an extremely thin a-Cr-Si-O passivation film at the surface of the *a*-Cr${}_{1-x}$Si${}_{x}$ film as well as by the p-d bonds at the phase boundaries (as discussed in [26], Section 2 A therein). Close to $x_{s}$, the crystallization temperature is $T_{k}\approx 550$ K or a little higher (depending on the annealing time), and thus a practical application of an *a*-Cr${}_{1-x}$Si${}_{x}$ film as a SRM up to temperatures T≈500 K seems to be reasonable.

The method for designing SRM’s proposed in the present paper has three advantages: (i) A SRM is now available with 0 μV/K absolute above superconducting temperatures and thus can also be applied at room temperature or even above, (ii) the assumption of reversible processes [which is the basis for Equation (A1)] is no longer necessary, and (iii) now we have the possibility to determine the thermopower very precisely by a method which is completely independent of the classical method (applied by Borelius et al. [27,28]). Regarding the advantage (iii) we state, when once a SRM with exactly 0 μV/K absolute is designed for one material, then the Thomson coefficient can be measured for all other interesting materials using Equation (A1). These measuring results can be compared directly with the experimental data of the Thomson coefficient of any other material. In other words, with this new SRM we have the possibility to check the question mentioned earlier, to what extent the processes acting on a real measurement of the Thomson coefficient τ (described in the Appendix A) are actually reversible, because Equation (A1) holds only for the case that only reversible processes act.

We close by a short statement on the formulas Equations (2) and (3) in connection with Equation (10) applied for the present calculations: The BTE formula $S_{i}^{0}$, Equation (3) is assumed to describe exclusively the scattering contribution to $S_{i}$. The correctness of this assumption is, however, not yet confirmed. The question of whether or not in $S_{i}^{0}$ the effect of “$\partial \mu_{i}^{0}/\partial T$” (respectively “$-\frac{\pi^{2}k_{B}^{2}T}{6E_{F,i}}$”) is contained indirectly is still open (see the discussion in [10]). However, this question does not influence the conclusions of the present paper, especially that the lower kink of S(±) at the discontinuity would occur at S(−)=S(+)=0. If in $S_{i}^{0}$, Equation (3), the effect of “$\partial \mu_{i}^{0}/\partial T$” (respectively “$-\frac{\pi^{2}k_{B}^{2}T}{6E_{F,i}}$”) would be contained indirectly, the S(±) vs. $\upsilon_{B}$ curves would be shifted in Figure 2, Figure 3 and Figure 4; however, in this case the discontinuity would also occur at S(−)=S(+)=0.

## 5. Conclusions

Applying the EMT to composites with different kinds of carriers in the different phases (electrons and holes), it is concluded that a discontinuity can be expected in the concentration dependence of the Seebeck coefficient which coincides exactly with the transition from negative values to positive ones. Such a dicontinuity (kink) is actually found experimentally in *a*-Cr${}_{1-x}$Si${}_{x}$ thin films sputtered from different Cr${}_{1-x}$Si${}_{x}$ targets representing a composite with the phases *a*-Cr${}_{3}$Si and *a*-Si. This feature can be applied to make reference standards for an absolutely zero Seebeck coefficient at room temperature and even at higher temperature. The experimental confirmation of this feature for *a*-Cr${}_{1-x}$Si${}_{x}$ alloys supports the theory of composites developed in the last years [3,9,10,19,26]. Under certain conditions such a kink at S=0μV/K can also be expected in the temperature dependence of *S*.

The experimentally finding that the electronic properties of amorphous Cr-Si films can differ fundamentally depending on the prevailing fabrication conditions suggests that the structure realized in any alloy is not only determined by the diffusion mobility of the atoms and that for this structure a (relative) minimum of the Gibbs’ free energy exists, but it depends also on the fact whether this structure is compatible with the demand that (spatial) continuity of the entropy and energy flux densities and their gradients is saved during the solidification process.

## Data Availability

The data that support the findings of this study are available on reasonable request to J.S.

## Symbols

*E*	Energy density (Ws/cm${}^{3}$)
*U*	Internal Energy density (Ws/cm${}^{3}$)
S	Entropy density (Ws/cm${}^{3}$T)
*t*	time (s)
*T*	temperature (K)
*S*	Seebeck coefficient (μV/K)
τ	Thomson coefficient (μV/K)
σ	electrical conductivity ($\Omega^{-1}{\mathrm{cm}}^{-1}$)
$\kappa_{e}$	electronic contribution to the thermal conductivity (Wcm${}^{-1}$K${}^{-1}$)
$S_{i}$, $\sigma_{i}$, $\kappa_{e,i}$ and $\upsilon_{i}$	are the Seebeck coefficient, electrical conductivity, electronic contribution to the thermal conductivity, and volume fraction, respectively, of the individual phase *i* [*i* = *A*, *B*]
$\alpha_{i}(=S_{i}^{0})$ and α (μV/K)	the “scattering terms” of the Seebeck coefficient in the phase *i* and the composite, respectively. The difference to *S* and $S_{i}$ is defined in the Appendix B.
$E_{F,i}$	the Fermi energy in the phase *i* (eV)
$L_{i}$	mean free path of the electronic carriers in the phase *i* (nm)
φ, $\varphi_{s}$	electrostatic potential (V/cm${}^{3}$)
$\mu_{i}^{0}$	chemical potential in the phase *i* (eV)
μ	electrochemical potential of the composite (eV)
*n*	electron densitiy [in a two-phase composite *n* is the electron densitiy in the phase with the higher potential (≡ phase *A*)] ($10^{22}$ cm${}^{-3}$)
*p*	hole densitiy in the phase *B* ($10^{22}$ cm${}^{-3}$)
$\zeta =\upsilon_{B}/\upsilon_{A}$	
β	a constant for a given alloy, which is determined by the average
	potential difference between the two phases.
$E_{c}$	band edge of the conduction band (eV)
*d*	average atomic distance (nm)
$N_{i}$	atomic density in the phase *i* ($10^{22}$ cm${}^{-3}$)
$x_{i}$	atomic concentration of the phase *i*
$x_{s}$	concentration, where the discontinuity occurs
$J_{i}$	electric current density in the phase *i*
$J_{Q,i}$	thermal current density in the phase *i*
$J_{S,i}$	entropie flux density in the phase *i*

## Appendix A. Seebeck Coefficient Standard Reference Materials

Absolute values of the thermoelectric power (Seebeck coefficient) of any material cannot be measured directly. It is always differences between two materials which are measured. That is why, in practice, Seebeck coefficient Standard Reference materials (SRM’s) are designed which allow to measure the absolute Seebeck coefficient of any material by a direct comparison with it.

SRM’s used in practice to measure the absolute thermopower of materials are generally not very accurate. An exception are superconductors, whose thermopower is exactly S=0μV/K. However, these can only be used for temperatures *T* smaller than the critical one $T_{c}$. For higher temperatures special SRM’s are used, which are, however, only approximations for an absolute value of *S*, which are the worse the higher the desired comparison temperature or reference temperature. Examples for such SRM’s are Pb, Sn or a special silver-normal-alloy (Ag + 0.37at%Au) (Borelius et al. [27,28,29], Christian et al. [30]). For this last strategy the fact was used that for a certain material the absolute Seebeck coefficient *S* is related with τ, the Thomson coefficient, by (first Kelvin relation)

(A1) $$S=\int_{0}^{T}\frac{\tau }{T}dT,$$

where *T* is the absolute temperature. The precision of such a SRM is however limited: First, Equation (A1) holds only if all the processes involved are reversible, but it is not yet completely clear to what extent this condition is fulfilled under the conditions of real measurement of τ. Second, the precision of the measured Thomson coefficients is restricted, because there are a series of sources for systematic uncertainties in measuring the Thomson coefficient τ (see, for instance Borelius et al. [27]).

Another practical method for supplying a SRM has been realized by Lowhorn et al. [31,32]. By two different, independent experimental techniques Lowhorn et al. have developed a special SRM (Te-doped Bi${}_{2}$Te${}_{3}$) which is designed as a comparison material for thermoelectric research in different laboratories. For this SRM uncertainties in the Seebeck coefficient are given by Lowhorn et al. [31] in the range of ≈1 *…* 6 μV/K in the temperature range T=10… 391 K. It remains a problem that absolute values for the Seebeck coefficient can only be measured relatively imprecisely, especially at room temperature and at higher temperatures.

## Appendix B. Comparison with Earlier EMT Formulas

There are a series of thermopower formulas published in the scientific literature, [33,34,35,36,37], which all are different from our formula Equation (1), and they do not provide such a discontinuity in the α versus *x* dependence. On the first look all these formulas look different. However, comparing their formulas in detail, all provide an identical concentration dependence ([23], Section 2.5.1. therein), α versus *x*, and what is especially remarkable, all these formulas contain $\alpha_{i}$, $\sigma_{i}$ and $\kappa_{i}$. Reason for this fundamental difference to the formula Equation (1) is the fact, that the authors start with an approximation of the thermal current density, $J_{Q}$, for instance Webman et al. [33],

(A2) $$J_{Q}=-\kappa \cdot gradT+\alpha \cdot \sigma \cdot grad\varphi .$$

κ and $\kappa_{i}$ are the total thermal conductivities in the composite and the phase *i*, respectively. α and $\alpha_{i}$ are the Seebeck coefficients in the composite and the phase *i*, respectively. (Why we distinguish between *S* and α becomes clear further below.) In formula Equation (A2) the quadratic term in α and the contribution of the chemical potentials of the phases, $\mu_{i}^{0}$, are not contained. An additional difference is that $J_{Q}$, Equation (A2), represents the total thermal current density (containing both the electronic part and the lattice part). However, if we leave aside this difference with respect to κ, we are still left with the fundamental difference that neglecting the quadratic term of α results in all three transport coefficients, $\alpha_{i}$, $\sigma_{i}$ and $\kappa_{i}$, appear in the EMT formula. This is not the case if the quadratic term in α is considered. If this point and the contribution of the chemical potentials are considered in $J_{Q,i}$ (formula (16) in [3]), then in the EMT formula only $\kappa_{e,i}$ and $\alpha_{i}$ occur. Starting with $J_{i}$ and $J_{Q,i}$ and correcting these inaccuracies contained in Equation (A2), then it follows for the EMT formula

(A3) $$\sum_{i}\upsilon_{i}\frac{\frac{\kappa_{e,i}}{\alpha_{i}-\left(d\mu_{i}^{0}/dT\right)/\left|e\right|}-\frac{\kappa_{e}}{\alpha -\langle (d\mu_{i}^{0}/dT)\rangle /\left|e\right|}}{\frac{\kappa_{e,i}}{\alpha_{i}-\left(d\mu_{i}^{0}/dT\right)/\left|e\right|}+2\frac{\kappa_{e}}{\alpha -\langle (d\mu_{i}^{0}/dT)\rangle /\left|e\right|}}=0,$$

(formula (30) in [3]), where $J_{i}$ and $J_{Q,i}$ are the local electric current density and the local thermal current density (without the lattice contributions), respectively, of the phase *i*. The angular brackets in Equation (A3), $\langle \ldots \rangle$, characterize an average.

Equation (A3) looks very complicated, especially because of the term $\langle \ldots \rangle$. A simplier formula we get, if $J_{i}$ and $J_{S,i}$ is used as the starting equations ([19]), where $J_{S,i}$ is the entropie flux density in the phase *i*:

(A4) $$\sum_{i}\upsilon_{i}\frac{\frac{\kappa_{e,i}}{\alpha_{i}+\left(d\mu /dT\right)/\left|e\right|}-\frac{\kappa_{e}}{\alpha +\left(d\mu /dT\right)/\left|e\right|}}{\frac{\kappa_{e,i}}{\alpha_{i}+\left(d\mu /dT\right)/\left|e\right|}+2\frac{\kappa_{e}}{\alpha +\left(d\mu /dT\right)/\left|e\right|}}=0$$

In Equation (A4) $d\mu_{i}^{0}/dT$ (which are different for the different phases) do not occur, insteadly the common dμ/dT.

Replacing $\alpha_{i}+\left(d\mu /dT\right)/\left|e\right|$ and $\alpha +\left(d\mu /dT\right)/\left|e\right|$ by $S_{i}$ and *S*, respectively, we get Equation (1), i.e., that $S_{i}^{0}$ in Equation (2) is identical with $\alpha_{i}$. This replacing was the decisive step for geting the final formula Equation (1). In the earlier practice of thermoelectricity, α was generally used as the thermopower, respective Seebeck coefficient, of a material, where the “thermodynamic term” $\left(d\mu /dT\right)/\left|e\right|$ did not occur in all the earlier thermopower formulas. This decisive step α⟶S, respective $\alpha_{i}⟶S_{i}$, i.e., the introduction of the additional “thermodynamic term” $\left(d\mu /dT\right)/\left|e\right|$ to the thermopower formulas founded in [10] has important consequences. In [10] it is also shown, that this contribution gives the answer on the question unsolved until now, why there are simple metals with positive thermopower as Cu, Ag, Au, and Li.

The difference between Equations (A4) and (A3) reflects a general problem for multi-band systems or multiphase systems: Applying $J_{i}$ and $J_{Q,i}$, problems can arise, which can be avoided when $J_{i}$ and $J_{S,i}$ is used. ([24], pages 28 and 40). So, Equation (A4), respective Equation (1), can be considered as the exact EMT formula under the special conditions (“the phase grains are spherical without preferred orientations and arranged in a symmetrical fashion”.)

In the formulas Equation (A4), Equations (A3) and (1) only the electronic part of the thermal conductivity occurs, but not the lattice part. This is in agreement with the seminal work of Harman and Honig [24], especially formula (1.17.1a) on page 40 and the explanatory text. In the electronic transport equations the lattice thermal conductivity does not appear.
